# Advances in the Role of Leucine-Sensing in the Regulation of Protein Synthesis in Aging Skeletal Muscle

**DOI:** 10.3389/fcell.2021.646482

**Published:** 2021-04-01

**Authors:** Yan Zhao, Jason Cholewa, Huayu Shang, Yueqin Yang, Xiaomin Ding, Qianjin Wang, Quansheng Su, Nelo Eidy Zanchi, Zhi Xia

**Affiliations:** ^1^Exercise Physiology and Biochemistry Laboratory, College of Physical Education, Jinggangshan University, Ji’an, China; ^2^Department of Exercise Physiology, University of Lynchburg, Lynchburg, VA, United States; ^3^School of Sport Medicine and Health, Chengdu Sport University, Chengdu, China; ^4^Hubei Provincial Collaborative Innovation Center for Exercise and Health Promotion, College of Health Science, Wuhan Sports University, Wuhan, China; ^5^Department of Physical Education, Federal University of Maranhão (UFMA), São Luís-MA, Brazil; ^6^Laboratory of Cellular and Molecular Biology of Skeletal Muscle (LABCEMME), São Luís-MA, Brazil

**Keywords:** age-related sarcopenia, anabolic resistance, protein synthesis, leucine-sensing, leucine response

## Abstract

Skeletal muscle anabolic resistance (i.e., the decrease in muscle protein synthesis (MPS) in response to anabolic stimuli such as amino acids and exercise) has been identified as a major cause of age-related sarcopenia, to which blunted nutrition-sensing contributes. In recent years, it has been suggested that a leucine sensor may function as a rate-limiting factor in skeletal MPS via small-molecule GTPase. Leucine-sensing and response may therefore have important therapeutic potential in the steady regulation of protein metabolism in aging skeletal muscle. This paper systematically summarizes the three critical processes involved in the leucine-sensing and response process: (1) How the coincidence detector mammalian target of rapamycin complex 1 localizes on the surface of lysosome and how its crucial upstream regulators Rheb and RagB/RagD interact to modulate the leucine response; (2) how complexes such as Ragulator, GATOR, FLCN, and TSC control the nucleotide loading state of Rheb and RagB/RagD to modulate their functional activity; and (3) how the identified leucine sensor leucyl-tRNA synthetase (LARS) and stress response protein 2 (Sestrin2) participate in the leucine-sensing process and the activation of RagB/RagD. Finally, we discuss the potential mechanistic role of exercise and its interactions with leucine-sensing and anabolic responses.

## Introduction

In 2020, the population over 60 years old in China was in excess of 250 million and accounted for 18.1% of the total population, becoming the first country in the world with an elderly population in excess of 100 million. Among them, the number of disabled and partially disabled elderly (i.e., core activity restrictions in self-care and mobility, such as feeding, dressing, transfer from bed, getting around inside the home, and bathing) exceeds 40 million. Age-related sarcopenia is a common disease in the elderly, and it is also a major predictor of disability. Sarcopenia is operationally defined as low muscle strength and is considered severe when low muscle quantity, quality, and low muscular strength are all present (Cruz-Jentoft et al., 2019). Age-related sarcopenia is characterized by the progressive decline of skeletal muscle mass and resultant impairment of physical and physiological function (Csete, 2021). With the accelerated growth of the older population, age-related sarcopenia will place an increasing burden on economic and social resources. Presently, there are no recognized consensus standards or guidelines for the clinical treatment of senile muscular atrophy, and no pharmacological interventions fully mitigate its occurrence and development (Chen et al., 2020). Therefore, clarifying the pathophysiological mechanisms of this disease and identifying potential intervention targets is important to promote the development of clinical intervention programs and new therapeutic drugs.

The maintenance of skeletal muscle mass depends on the dynamic balance between the rate of muscle protein synthesis (MPS) and degradation (MPB). The algebraic difference (MPS minus MPB) between the two indicates the state of net protein balance (NPB) (Tipton and Phillips, 2013; Murach and Bagley, 2016). When NPB is positive, protein deposition increases, which eventually induces muscle hypertrophy; on the contrary, when NPB is negative, protein deposition decreases and atrophy occurs (Holwerda et al., 2019). The decline in skeletal muscle mass as a result of age is primarily due to a decrease in MPS in response to anabolic stimuli such as amino acids and exercise, also referred to as anabolic resistance (Rong et al., 2020). Thus, how to effectively ameliorate the decline in NPB and protein deposition of aging skeletal muscle has become an important area of research in biomedical sciences. Exercise can enhance the sensitivity of aging skeletal muscle to dietary amino acid and protein and thus has important potential in promoting skeletal muscle protein deposition (Rebelo-Marques et al., 2018; Ebner et al., 2020). Previous studies have confirmed that resistance or aerobic exercise combined with dietary protein and/or amino acid ingestion induces greater MPS rates, hypertrophy and muscular performance, than nutritional support alone (Xia et al., 2016; Phillips and Martinson, 2019). Leucine, an essential amino acid (and one of the three branched-chain amino acids), is recognized as a key anabolic stimulus and can be directly involved in the regulation of MPS both on a substrate and signal transduction level. Additional studies have shown that leucine sensing has an important effect on MPS in aging skeletal muscle (Devries et al., 2018; Lehman and Abraham, 2020).

As previously discussed, anabolic resistance is a key factor in age-related sarcopenia, and leucine sensing and response play an important role in the regulation of MPS. Therefore, enhancing the sensing and the response of aging skeletal muscle to anabolic stimuli such as leucine to counteract anabolic resistance is an important and timely topic of research in the field of aging. This paper, therefore, will review the latest research to explore new mechanisms of age-related sarcopenia and new strategies for its prevention and treatment and provide theoretical rationale for the application of related targets in clinical translational medicine.

## Important Signal Molecules Involved in Leucine-Mediated Protein Synthesis in Skeletal Muscle

### Mechanistic/Mammalian Target of Rapamycin Complex 1: The Coincidence Detector for Protein Synthesis

In higher eukaryotes such as mammals, there is an evolutionarily conserved phosphatidylinositol three-kinase-related kinase family member mechanistic/mammalian target of rapamycin (mTOR). As a core component (catalytic subunit), mTOR participates in the formation of two multisubunit protein complexes, termed mTORC1 and mTORC2. In terms of the regulation of protein synthesis, mTORC1 mediates translation regulation via phosphorylating two downstream substrates S6 kinase 1 (S6K1) and eukaryotic translation initiation factor 4E-binding protein 1 (4E-BP1).

**FIGURE 1:**
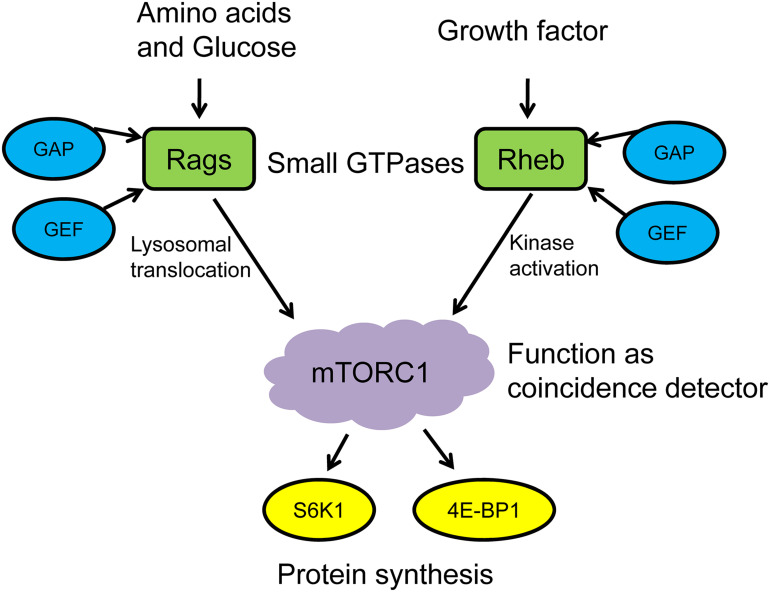
mTORC1 functions as a coincidence detector to regulate protein synthesis. GAP, GTPase-activating protein; GEF, GMP exchange factor; Rags, ras-related GTPases; Rheb, ras homolog enriched in brain; mTORC1, mechanistic/mammalian target of rapamycin complex 1; S6K1, S6 kinase 1; 4E-BP1, eukaryotic translation initiation factor 4E-binding protein 1.

mTORC1 is located in the cytoplasm in its inactive form. The activation of mTORC1 generally includes two steps: (1) mTORC1 translocates from the cytoplasm to the surface of lysosomes in response to environmental stimuli and (2) the mTORC1 recruitment complex and activator colocalize on the surface of lysosome, which then is activated under the premise of satisfying the activation conditions. It should be noted that these activation conditions are strict, which means mTORC1 can only be activated when both nutrients and growth factors are present (Sancak et al., 2008; Sabatini, 2017; Lehman and Abraham, 2020). Therefore, mTORC1 works as the “coincidence detector” to detect these two activation conditions. On the one hand, nutritional signals such as amino acids and glucose promote the conversion of Ras-related GTPases (Rag GTPases) to active state via the effects of GTPase-activating protein (GAP) and guanine nucleotide exchange factor (GEF), then induce the lysosomal translocation of mTORC1. On the other hand, growth factors and cellular stress signals regulate the nucleotide loading states of ras homolog enriched in brain (Rheb), a small GTPase on the lysosomal surface, which directly stimulates the functional activity of mTOR kinase when binding with GTP and then phosphorylates the downstream targets S6K1 and 4E-BP1 (Sabatini, 2017; Condon and Sabatini, 2019). Leucine promotes MPS via mTORC1 activation, and its effects are more pronounced than other essential amino acids. Leucine has also been suggested to decrease muscle proteolysis and, when combined with exercise, provides a synergistic MPS response (Zanchi et al., 2008; Xia et al., 2017a). Thus, it can be seen that the activation of mTORC1 and the execution of its “coincidence detector” that function under the regulation of small GTPases are critical for protein synthesis in skeletal muscle (Figure 1).

### Key Upstream Regulator of mTORC1: Small GTPase

#### Rag GTPases

At present, several amino acids have been shown to stimulate mTORC1 activation, and their signal transduction involves effects mediated by distinct small GTPases (Zhu and Wang, 2020). In understanding the regulation of amino acids on the mTORC1 pathway, a milestone was the recognition of Rag GTPases that mediate amino acid signal transduction to mTORC1. Mammalian skeletal muscles have four Rag proteins: RagA and RagB share almost 90% protein sequence identity and are functionally redundant, whereas RagC and RagD share about 81% sequence identity and are also redundant (Schurmann et al., 1995; Sekiguchi et al., 2001). As early as 2008, Sancak et al. (2008) and Kim et al. (2008) confirmed that Rag GTPases could input amino acid signals into the mTORC1 signaling pathway and induce downstream molecular events by using biochemical purification and RNAi technology, respectively. These recent studies further found that RagB forms a heterodimer with RagD via sharing their roadblock domain at the C-terminus, and this dimerization is imperative for mTORC1 activation in response to leucine stimulation (Lee et al., 2018).

The *in vitro* studies showed that the GTP-/GDP-loading status of Rag GTPases controls its enzymatic activity and regulates the localization and activation of mTORC1 on the lysosome membrane (Bar-Peled et al., 2012; Meng and Ferguson, 2018). When amino acids are sufficiently present, RagA/B is bound to GTP, and RagC/D to GDP, which enhances the interaction between the heterodimeric Rag GTPase complex and Raptor and promotes mTORC1 translocation to the lysosome surface for subsequent activation. It must be pointed out that RagB^GTP^/RagD^GDP^ play a central role in the mTORC1 signaling pathway in response to leucine stimulation (Lee et al., 2018). Conversely, under conditions of amino acid starvation, RagA/B loaded with GDP and RagC/D loaded with GTP will inactivate the heterodimeric Rag GTPase complex and inhibit the activation of mTORC1 (Kim et al., 2008; Sancak et al., 2008). Therefore, the nucleotide status of Rag GTPases is strictly controlled by amino acid availability.

However, it is important to note that human experiments have produced slightly different results. Recent experiments by Hodson et al. (2017) showed that mTOR is highly colocalized with LAMP2 (lysosomal marker) in the basal state (10 h overnight fasting and 7 days of exercise cessation). Upon protein-carbohydrate feeding and resistance exercise stimulus, mTOR/LAMP2 complexes translocated to the cell periphery. Hodson and Philp (2019) proposed that this translocation is a key event in mTORC1 activation after resistance exercise and amino acid ingestion in human skeletal muscle. Similar conclusions have been drawn by Abou Sawan et al. (2018), who investigated postprandial myofibrillar protein synthesis at rest and after endurance exercise in young adults. Further research is necessary to explore the relevance of mTORC1 colocalization in the aging skeletal muscles.

#### Rheb GTPase

Rheb, a member of the Ras superfamily, is widely expressed in various tissues including skeletal muscle and resides at the lysosomal surface by its last 15 amino acids, which contain a C-terminal CAAX box (Sancak et al., 2010; Menon et al., 2014). As shown in Figure 1, although Rag GTPases play an important role in enhancing translocation of mTORC1 to lysosome and subsequent activation, they cannot activate mTORC1 directly. In other words, this process also involves the role of another small GTPase Rheb. Works from Sancak’s research group showed that mTORC1 rapidly translocated to the lysosome in response to amino acid stimulation and was activated by Rheb. Although RNAi-induced suppression of Rheb expression inhibited mTORC1 activation, it could not interfere with the lysosomal translocation after amino acid stimulation (Sancak et al., 2008, 2010).

After mTORC1 is recruited to the lysosomal surface by Rag GTPase heterodimers, Rheb works as the key activator to bind directly with the HEAT (N-heat and M-heat) and FAT domains of mTOR via the switch II region under conditions of amino acid repletion (Roccio et al., 2006; Yang et al., 2017). The inputs of this signal toward mTORC1 are integrated via Rag GTPases rather than changing the nucleotide loading status of Rheb in response to amino acid stimulation. The interaction between switch II region and the domains of mTOR was not affected by GTP itself. However, the GTP-bound Rheb (active state) is still required for mTORC1 activation (Sancak et al., 2008).

Taken together, the GTP-/GDP-loading status of both RagB/RagD heterodimer and Rheb GTPase may be the rate-limiting factor in mTORC1 activation induced by leucine stimulation, and a series of protein and/or complexes that are able to regulate their nucleotide state by working as their GAP and/or GEF may play the central role.

### Regulation of Nucleotide Loading Status of Rag and Rheb GTP Enzymes

In addition to the interactions between Rheb, Rag GTPases, and mTOR, several protein complexes have been found to act upstream of Rheb and Rag GTPases to control their nucleotide loading status: either by promoting the hydrolysis of GTP and increasing the ratio of GDP/GTP by working as the GAP, or by stimulating the exchange between GDP and GTP by working as the GEF (Cherfils and Zeghouf, 2013).

#### Ragulator Complex

Due to the lack of a lipid-targeting signal, the protein complex named Ragulator is needed to help Rag GTPases locate on the surface of lysosome and then regulate mTORC1 translocation. Ragulator is a pentameric complex composed of p18, p14, MP1, C7orf59, and HBXIP (also called LAMTOR1-LAMTOR5), in which p14/MP1 form a heterodimer with C7orf59/HBXIP, respectively, and are encapsulated by p18. Rag GTPases network directly with p14/MP1 heterodimer directly via its C-terminal domain, while Ragulator anchors the Rag GTPases to the lysosomal surface through myristoylation and palmitoylation at the N-terminus of p14 (Sancak et al., 2010; de Araujo et al., 2017).

In addition to the role of localizing Rag GTPase to the lysosome, Ragulator is also involved in the regulation of Rag GTPase activity by functioning as its GEF. In terms of leucine-stimulated mTORC1 activation, Ragulator works as the GEF for RagB and promotes GDP release from RagB^GDP^ and subsequent GTP binding and thus led the conversion from the pre-active heterodimer RagB^GDP^/RagD^GDP^ to active RagB^GTP^/RagD^GDP^ (Bar-Peled et al., 2012). Moreover, Ragulator has also been shown to serve as the GEF for both RagA (Bar-Peled et al., 2012) and RagC (Shen and Sabatini, 2018), which allows them to become GTP loaded and thereby play a role in other amino acid-induced mTORC1 signaling.

#### GATOR Complex

GTPase-activating protein activity toward the Rag GTPases (GATOR) complex is an octamer composed of GATOR1 and GATOR2. GATOR1 has three stably interacting subunits: DEP domain-containing 5 (DEPDC5), nitrogen permease regulator 2-like protein (Nprl2), and Nprl3, while GATOR2 consists of meiosis regulator for oocyte development (Mios), Sec13, Sec13-like protein (Seh1L), WD repeat-containing protein 24 (WDR24), and WDR59. Functionally, GATOR1 negatively regulates mTORC1 activation, whereas GATOR2 is a positive regulator for mTORC1 signaling and acts either upstream of or in parallel to the GATOR1 (Bar-Peled et al., 2013; Wolfson et al., 2016).

GATOR1 binds RagB and functions to inactivate it by hydrolyzing the GTP to GDP, thus working similar to the GAP of RagB to inhibit the leucine-sensing pathway. This GAP mode is implemented by RagB binding to the Nprl2 and/or Nprl3 subunits of GATOR1 (Bar-Peled et al., 2013). Recent studies have indicated that GAP also modulates RagB activity in an inhibitory mode, which is achieved by the DEPDC5 subunit of GATOR1 binding to RagB (Shen and Sabatini, 2018). More recently, Graber et al. (2019) have observed that specific knockout of DEPC5 in skeletal muscle of adult mice attenuates GATOR1 inhibition of mTORC1, resulting in soleus muscle hypertrophy.

The interaction between GATOR1 and RagB is controlled by many regulatory proteins and/or complexes. Of these, KPTN-, ITFG2-, C12orf66-, and SZT2-containing regulator of TOR (KICSTOR) works as the scaffold complex for GATOR1 to localize on the lysosomal surface and thus is important for leucine-induced mTORC1 signaling (Wolfson et al., 2017). Deletion of any part of KICSTOR will result in the mislocalization of GATOR1, and it renders cells insensitive to amino acid starvation. For example, deletion of SZT2 will lead to persistent mTORC1 activation upon amino acid starvation in many tissues such as muscle, and the overexpression of DEPDC5 protein, a GATOR1 subunit, will partly ameliorate this unfavorable change (Peng et al., 2017; Wolfson et al., 2017). It can be observed that the KICSTOR complex is able to strengthen the interaction between GATOR1 and RagB, thereby negatively regulating mTORC1 activation. However, it is unclear whether it has other roles in modulating GATOR1 activity. Conversely, a recent study by Pal et al. (2018) showed that Src tyrosine kinases produce positive effects on amino acid-induced mTORC1 signaling by disrupting GATOR1-Rag GTPase interaction. However, the potential role of Src in response to leucine stimulation remains undefined.

#### FLCN Complex

The folliculin (FLCN) complex consists of protein FLCN and FLCN-interacting proteins 1 and 2 (FNIP1/2). A previous study suggested that this complex can be involved in both mTOR and AMPK signaling (Baba et al., 2006), while recent results demonstrated that it translocated to the lysosomal surface and bound with Rag GTPases in response to amino acid stimulation. FLCN also works as the positive regulator in mTORC1 activation by hydrolyzing the GTP to GDP of RagC and RagD (Petit et al., 2013; Tsun et al., 2013). Given the crucial role of GDP-bound RagD in leucine-induced mTORC1 activation, the FLCN complex is possibly involved in this process, but this speculation awaits further experimentation.

#### TSC Complex

TSC1 and TSC2 (tuberous sclerosis complex), along with TBC1D7, form a functional complex (the TSC protein complex). Rheb is negatively regulated by the TSC complex through the GAP activity of TSC2, and mTORC1 cannot be further activated. In contrast, the GTP-bound form of Rheb is able to activate mTORC1 by allosterically re-aligning residues in the active site (Yang et al., 2017).

The TSC complex is generally involved in the modulatory role of growth factors on Rheb activity by acting as a downstream effector of phosphatidylinositol 3-kinase (PI3K) and protein kinase B/Akt. This pathway mediates TSC2 phosphorylation, then results in the dissociation of the TSC complex from the lysosome, Rheb activation, and subsequent mTORC1 activation (Menon et al., 2014). However, it has also been found that Rheb can be activated via TSC2 phosphorylation in a PI3K-independent manner in response to eccentric contractions (Jacobs et al., 2013). Under energy depletion, AMP-activated protein kinase (AMPK) promotes Rheb-TSC interaction by phosphorylating TSC2 and Raptor, thereby inhibiting mTORC1 activation and cell growth (Inoki et al., 2003; Gwinn et al., 2008).

The modulation of the TSC complex on Rheb-mTOR is generally considered to be necessary for mTORC1 activation when responding to amino acid stimulation. Arginine cooperates with growth factor signaling, which further promotes dissociation of TSC2 from lysosomes and subsequent activation of Rheb and mTORC1, as reported by Carroll et al. (Carroll et al., 2016). However, in terms of leucine stimulation, the latest report by Suryawan et al. found that neonatal pigs fed with a low-protein diet supplemented with leucine or its metabolite β-hydroxy-β-methylbutyrate (HMβ) failed to promote the Rheb–mTOR complex formation, but this effect was not observed in pigs fed a high-protein diet combined with leucine supplementation (Suryawan et al., 2020). The authors suggest that these results are in line with those of Yoshida et al. (2018) showing that leucine and HMβ do not engage mediators (such as Rheb) of the growth factor signaling pathway in mTORC1 activation. Thus, it is still necessary to find out whether the TSC complex is involved in the regulation of skeletal MPS induced by leucine supplementation as the GAP of Rheb.

## Rate-Limiting Factor in Leucine-Induced Skeletal Muscle Protein Synthesis: Leucine Sensor

mTORC1, as a coincidence detector of amino acids and growth factors, is highly sensitive to the changes of leucine levels under the regulation of small GTPases and their GAP and GEF. However, mTORC1 itself does not have the ability to sense the level of leucine in cells. In light of this information, there must be other proteins performing functions as the leucine sensor upstream of mTORC1 in the regulation of skeletal MPS. At present, two leucine sensors have been identified, namely, leucyl tRNA synthetase (LARS) and stress response protein 2 (Sestrin2).

It should be noted that most of the information discussed in this section is derived from cell culture data. Differences in conditions such as oxygen, glucose, insulin, and amino acids levels must all be taken into consideration when interpreting the results of these studies. Therefore, although these data provide a useful reference for discussion, they are not directly inferable to muscle and clinical scenarios until future research in the field of clinical and translational medicine is conducted.

### LARS

**FIGURE 2:**
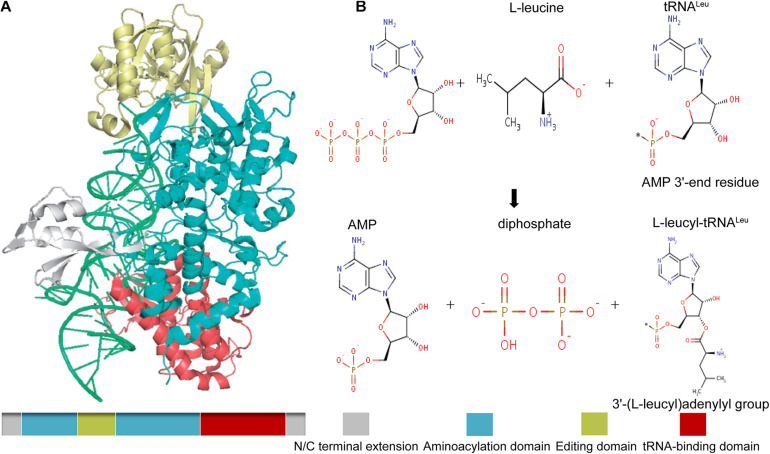
Schematic drawing of the structure of mammalian LARS **(A)**, and its canonical catalytic function **(B)**. Gray, N/C terminal extension; green, aminoacylation domain; gold, editing domain; red, tRNA-binding domain. The canonical catalytic function of LARS including tRNA-independent pre-transfer editing, tRNA-dependent pre-transfer editing, and post-transfer editing.

In mammals and other higher eukaryotes, LARS has a molecular weight of 134 KDa and contains an extended domain at the N- and C-terminal end, a tRNA-binding domain near the C-terminal end to identify and bind its specific tRNA, and an aminoacylation domain for the activation and transfer of amino acids. In addition, it also has a connecting polypeptide (CP1) domain, which is inserted into the synthetic active site as the editing and correcting active site, which is used to check and hydrolyze the incorrectly aminoacylated tRNA (Figure 2A; Tukalo et al., 2005). As one of the aminoacyl-tRNA synthetases, LARS has received much attention because of its canonical function, that is, to catalyze the reaction of leucine with tRNA^Leu^ to produce L-leucyl-tRNA^Leu^, which provides raw materials for protein synthesis (Figure 2B). However, several studies have found that the positive effect of LARS on protein synthesis seems to be more attributable to its non-canonical function (Han et al., 2012; Segev and Hay, 2012).

Recently, we reviewed the role and potential mechanism of LARS in protein synthesis of aging skeletal muscle (especially the non-canonical function) (Xia et al., 2020). Han et al. (2012) and Bonfils et al. (2012) reported at the same time that in response to leucine stimulation, mTORC1 can be activated by LARS as a leucine sensor, and this process is mediated by Rag GTPases. Bonfils et al. (2012) found that Cdc60, the yeast homolog of LARS, interacted with GTR1 (yeast homolog of RagA/B) rather than GTR2 (yeast RagC/D) in a leucine-dependent manner, while a study in human embryonic kidney-293 cells by Han et al. (2012) showed that LARS binds and interacts with RagD. At present, there is consistent support for the research results from Han’s group. In the case of sufficient leucine, LARS realizes leucine sensing through its aminoacylation active site, specifically combines with RagD through the VC-b domain, and acts as the GAP of RagD, making it convert from the GTP-bound inactive form (RagD^GTP^) to the GDP-bound activated state (RagD^GDP^), and then triggers the activation of mTORC1 (Lehman and Abraham, 2020).

However, when the glucose content in the medium was controlled, the leucine-sensing function of LARS significantly decreased, suggesting that glucose may be an essential environmental factor for LARS to regulate mTORC1 (Yoon et al., 2020). This finding further highlights the important role of small GTPases, especially Rag and Rheb, in leucine-stimulated mTORC1 activation and subsequent protein synthesis (Figure 1). Most recently, Suryawan et al. (2020) observed that the association between LARS and RagD was not affected by leucine. Therefore, it was further proposed that leucine regulates the Sestrin2–GATOR–Rags complexes to facilitate mTORC1 activation in neonatal muscle. Previous human experiments also showed that leucine could enhance mTOR phosphorylation but did not affect the LARS–mTORC1 complex (Carlin et al., 2014). Moreover, the recent research results from Sato et al. (2018) showed that the LARS small interfering RNA (siRNA) knockdown in C2C12 myotube cells did not affect cell hypertrophy, although si-LARS successfully knocked down gene and protein expression of LARS in differentiated myotubes. Similarly, si-LARS had no effect on mTOR phosphorylation, further suggesting that LARS may not be necessary for the activation of mTORC1. These observations might reflect a compensatory system for sensing leucine through other proteins or complexes, which function as intracellular sensors (such as Sestrin2).

D’Hulst et al. (2020) observed for the first time that the expression level of LARS in the vastus medialis muscle of elderly male and female subjects was lower than that of young subjects. Although the difference was not statistically significant (*P* = 0.11), the authors suggest that this result is due to the large variability between subjects. We hypothesize that LARS, as a potential leucine sensor and regulator of mTORC1 in skeletal muscle, is downregulated with aging, which may support the important role of LARS in age-related anabolic resistance and the occurrence and development of sarcopenia. However, in the future, large sample screening is still needed to effectively control the influence of variability between subjects, so as to confirm expression changes with aging.

### Sestrin2

There are three subtypes of Sestrins in mammals, namely, Sestrin1, Sestrin2, and Sestrin3, which are mainly regulated by environmental stimuli such as DNA damage, oxidative stress, and hypoxia. Among them, Sestrin2 is believed to play a central role in the activation of mTORC1 in response to leucine stimulation. Structurally, the leucine-binding site of Sestrin2 comprises a hydrophobic pocket flanked on each side by opposing positive and negative charges, respectively, contributed by Arg390 and Glu451, which make it well suited to accommodate leucine (Saxton et al., 2016).

Sestrin2, as a subtype of Sestrins, has been found to have the following characteristics that align with its assumed role as leucine sensor: (1) its affinity to leucine is consistent with the concentration of leucine sensed; (2) its mutants that do not bind leucine could not transmit the signal (the presence of leucine) to mTORC1; and (3) the deletion of Sestrin2 and its homologs makes the mTORC1 pathway insensitive to leucine deficiency (Wolfson et al., 2016). These findings clearly highlight the potential of Sestrin2 to participate in the regulation of the mTORC1 pathway as a leucine sensor. Specifically, it has been shown that Sestrin2 can bind to the GATOR2 subcomplex through its WDR24 and Seh1L subunits, inhibit the activity of the GATOR1 subcomplex (the negative regulator of mTORC1), and act as the GAP of RagA/B to promote the conversion of RagB^GTP^ to inactive RagB^GDP^. Therefore, Sestrin2 negatively regulates mTORC1 activity and is functionally antagonistic to LARS (Parmigiani et al., 2014; Kim et al., 2015).

It is important to note that the Michaelis constant (Km) of LARS for leucine activation is about 45 μM, while leucine binds Sestrin2 with a dissociation constant (Kd) of approximately 20 μM. This means that Sestrin2 binds leucine with an affinity (i.e., Kd) significantly lower than the leucine levels detected in fasted rats and humans (circulation: 129 vs 157 μM; muscle: 120 vs 167 μM). Therefore, if the leucine levels in muscle cells are similar to that detected in tissue extract, Sestrin2 will be completely saturated with leucine under physiological conditions (Xu et al., 2019). Thus, Sestrin2 was suggested to be the leucine sensor that binds GATOR2 only in the absence of leucine, and this interaction is proposed to inhibit mTORC1 activation (Lee et al., 2016). However, it is not clear whether LARS and Sestrin2 regulate the activation of mTORC1 independently or cooperatively. The recent studies showed that the expression of Sestrin2 in skeletal and cardiac muscle tissues decreases with aging (Lenhare et al., 2017; Quan et al., 2017). In view of the negative regulation of Sestrin2 on mTORC1 activation, if Sestrin2 plays an important co-regulatory role in the regulation of leucine sensing and protein metabolism in aging skeletal muscle, enhanced mTORC1 activity should be observed, but this is not the case currently. Thus, in our humble opinion, Sestrin2 and LARS may play a role in different growth stages (Xia et al., 2020). However, the binding of Sestrin2 to GATOR2 is leucine-dependent. When leucine is sufficiently present, leucine competes with Sestrin2 to bind GATOR2 and results in the dissociation of Sestrin2 and GATOR2. Subsequently, RagB^GTP^ fails to be converted to GDP-bound inactive form (RagB^GDP^), and the inhibition of mTORC1 is blocked (in line with the attenuated nutrient sensing in aging skeletal muscle) (Wolfson et al., 2016). Therefore, we speculate that Sestrin2 may also be a key factor in the process of sensing leucine stimulation and regulating protein synthesis via mTORC1 activation in aging skeletal muscle. On the other hand, HMβ has been shown to induce skeletal muscle hypertrophy by stimulating mTORC1. However, the latest evidence shows that it does not lead to the dissociation of Sestrin2 and GATOR2 subcomplex (Suryawan et al., 2020). This finding may suggest that HMβ-induced mTORC1 activation is not achieved by inhibiting Sestrin2, and the potential mechanism needs to be further studied. In order to avoid bias, it should be mentioned that there are two recent reports showing the protective effects of chronic mTORC1 inhibition on muscle mass, muscle function, and even the molecular changes associated with age-related sarcopenia (Joseph et al., 2019; Ham et al., 2020). In our humble opinion, further validation pertaining to the change of mTORC1 activity with aging is warranted under the premise of excluding hidden confounding factors (i.e., all other age-related diseases or phenomenon which might perturb mTORC1 activity).

Finally, although Sestrin1 is not an identified leucine sensor, its role in skeletal muscle needs to be highlighted. Most recently, Xu et al. (2019) revealed that Sestrin1 is expressed at significantly higher levels than Sestrin2 and Sestrin3 and exhibits the greatest sensitivity to leucine-induced dissociation from GATOR2. Thus, the authors suggested that the higher expression of Sesetrin1 in skeletal muscle engenders greater sensitivity of mTORC1 to activation at lower leucine levels. Zeng et al. (2017) previously reported that the expression of Sestrin1 was upregulated following an acute resistance exercise intervention, while Sestrin2 and Sestrin3 were not changed. Taking the effects of resistance exercise on MPS into consideration, we might have reason to investigate whether or not the upregulation of Sestrin1 postexercise enhances skeletal muscle sensitivity to leucine-induced mTORC1 activation.

## Potential Role of Exercise in Regulation of mTORC1 Signaling

There is, at present, no research directly investigating the regulation of skeletal muscle leucine sensing by exercise. However, as mentioned above, LARS and Sestrin2 can work as leucine sensors to directly participate in the mTORC1 activation of skeletal muscle in response to leucine stimulation. Moreover, the complexes of Ragulator, GATOR, FLCN, and TSC can also be involved, as the GAP and/or GEF of small GTPases Rag and Rheb. Theoretically, exercise may affect this series of proteins involved in the regulation of the nucleotide loading status of Rag and Rheb GTPases, thus regulating the leucine sensing and response of mTORC1. In other words, at least some of the intermediates of the leucine-sensing system also engage in mTORC1 regulation in response to exercise, and while these may be independent of leucine-sensing, they may function to bolster the leucine-sensing pathway.

### Effects of Exercise on Nucleotide Loading Status Regulatory Proteins of Rag and Rheb GTP Enzymes

To the best of our knowledge, there is no research that has investigated the effect of exercise on the complex of Ragulator and GATOR and its subunits. The FLCN complex functions as the GAP of RagC/D and positively regulates the activation of GTP in response to leucine stimulation by promoting the hydrolysis of the GTP to GDP. In the recent experiment by Collodet et al. (2019), wild-type zebrafish were forced to swim at a speed of 60 cm/s for 2 h and 40 min. The trunk muscles were separated 3 h after exercise, and the expressions of FLCN and FNIP2 genes were detected by qPCR. The results showed that the expression level of these two genes were significantly elevated above that of the control group. It should be noted that the protein expressions of these two genes were not determined and zebrafish is not a common species used to model exercise in muscle physiology. Therefore, it is difficult to draw a conclusion that exercise may enhance the GAP function of FLCN toward Rag GTPases. Further studies using rodents and eventually humans are required to clarify the interaction between exercise, FLCN complex, and mTORC1 activation.

Tuberous sclerosis complex 2, as the GAP of Rheb, inhibits the activity of mTORC1. In the research by Kim and Lee (2017), rats receiving a high-fat diet were trained by treadmill running at a speed of 15 m/min for 30 min in the first 4 weeks and progressed to 40 min and 18 m/min in the next 4 weeks. Immunofluorescence showed that the expression of TSC2 in metatarsal muscle of rats was significantly downregulated. The human experiment from Sawan’s group also observed that a 60-min training at 70% VO_2_ max intensity on a treadmill with 1% incline resulted in the dissociation of TSC2-Rheb in the vastus lateralis muscle and enhancement of the lysosomal co-localization of mTOR and Rheb after exercise (Abou Sawan et al., 2018), and the same results were found following acute resistance exercise by Song et al. (2017). Moreover, Jacobs et al. (2013) found that eccentric exercise could stimulate the lysosomal localization of mTOR and the dissociation of TSC2 from the lysosome in tibialis anterior muscle of mice. According to our experience, in rodent models such as rats and mice, it is difficult to distinguish between aerobic and resistance training-induced molecular signaling and adaptation, as all exercises likely result in an overt global stress response that robustly stimulates all key growth signals. In elderly humans, it is entirely possible that opposing exercise modalities “distract” from one another by producing conflicting molecular signals. In view of the fact that the human experimental studies by Abou Sawan et al. (2018) and Song et al. (2017) have observed consistent results, there seems to be reason to believe that both aerobic exercise and resistance exercise can downregulate the expression of TSC2, promote the dissociation of TSC2-Rheb, and enhance the lysosomal co-localization of mTOR-Rheb, which subsequently inhibits the conversion of Rheb to inactive state loaded with GDP and promotes the activation of mTORC1. However, the different results caused by different exercise intensity, frequency, and duration still need to be clarified by further research.

### Effects of Exercise on LARS and Sestrin2

We are also not aware of any direct research concerning the effect of exercise on the expression of LARS, with the focus of research mostly on Sestrin2. In the study by Crisol et al. (2018), C57BL/6J mice were used to evaluate the effects of acute aerobic exercise and chronic aerobic training on Sestrin2 gene expression and protein expression. Two and four hours following 60 min of acute treadmill exercise at 60% maximum load intensity, the quadriceps muscle was sampled. The results showed a trend toward upregulation, but without statistical significance. However, Sestrin2 expression was downregulated after 4 weeks of chronic aerobic training with the same exercise load and time (5 days/week). Conversely, two reports from Fu’s group showed a significant increase of Sestrin2 expression in the quadriceps of mice after a 6-week aerobic exercise training with increasing workloads—50% VO_2_ max for 20–30 min/day for the first week—then were increased to 75% VO_2_ max for 60 min/day (Liu et al., 2015; Wang et al., 2018).

In terms of the effects of resistance exercise, two human experimental studies detected Sestrin2 electrophoretic mobility following an acute and chronic exercise intervention. The abundance of the slowest-migrating δ form of Sestrin2 was taken as phosphorylation, while the expression of all forms of Sestrin2 (α, β, γ, and δ form) was taken as total protein. Of these, one study found increased abundance of a slower-migrating δ band of Sestrin2 at 2 h after acute resistance exercise, but the chronic intervention for 12 weeks (2 days/week) had no effect on it (Zeng et al., 2017). The other study observed an increased abundance of the δ band and the decrease of the γ band at 90 and 240 min following acute resistance exercise, an upregulation in gene expression 240 min after exercise, and no effect following the supplementation of milk protein concentrate (Zeng et al., 2018). According to the previous study in human embryonic kidney-293 cells by Kimball et al. (2016), the abundance of the δ band was negatively correlated with mTORC1 activation induced by leucine. Moreover, Luciano et al. (2017) compared the effects of a 12-week muscular endurance resistance training, strength resistance training, and hypertrophy resistance training by using rats. The results showed that the expression of phosphorylated mTOR^Ser2448^ and 4E-BP1^Thr37/Ser46^ in quadriceps of these three groups was significantly higher than that of the control group, and the expression level in the hypertrophy resistance training group was the highest, followed by strength resistance training group. Importantly, the expression level of Sestrin2 showed an inverse relationship to phosphorylated mTOR^Ser2448^ and 4E-BP1^Thr37/Ser46^.

These clues suggest that the results of the studies on the effect of exercise on Sestrin2 expression differed because of the differences between, e.g., exercise patterns, exercise programs, and species. At present, the exact effect of exercise on Sestrin2 expression cannot be confirmed, and the relationship between the changes of Sestrin2 expression and mTORC1 activity is not fully clear. In addition, it should be pointed out that there is no report on the effect of exercise on Sestrin2 expression in aging skeletal muscle. Although our previous study has shown that aerobic and resistance training combined with leucine supplementation can downregulate Sestrin2 expression and enhance mTOR phosphorylation in skeletal muscle of aging mice, the effect of exercise itself cannot be confirmed due to the confounding influence of leucine (Xia et al., 2017b).

## Conclusion and Prospects

**FIGURE 3:**
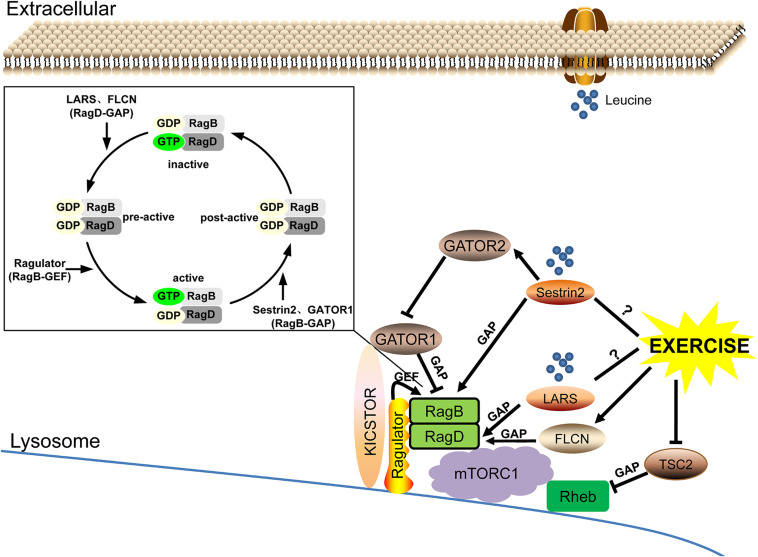
The leucine sensing signaling pathway upstream of mTORC1 and the potential role of exercise. ➞, activate; **⊣**, inhibit; ?, unknown mechanism.

As the rate-limiting factors of leucine-mediated protein synthesis in skeletal muscle, leucine sensors LARS and Sestrin2, and potentially Sestrin1, can regulate the nucleotide loading state of small GTPases RagB/RagD and Rheb by functioning as their GAP or GEF under the action of Ragulator, GATOR, FLCN, and TSC complexes and then regulating the response of mTORC1. Exercise may be involved in the regulation of leucine-sensing by upregulating the FLCN complex and inhibiting the expression of TSC2, but its effect on LARS remains to be explored and its exact effect on Sestrin2 needs to be further clarified (Figure 3).

The research on amino acid sensors is still in the early stages, and as far as leucine sensing is concerned, there are still many scientific issues to be explored. For example: (1) Are the identified leucine sensors LARS and Sestrin2 independently involved in MPS regulation at different growth stages or cooperatively integrated throughout the life cycle? Can either sense amino acids other than leucine and thus participate in the regulation of MPS? (2) How does exercise (different exercise types, intensities, frequencies, and durations) affect the activity of leucine sensors (especially LARS), and consequently the response of mTORC1? (3) Can the adaptive changes observed in rodent studies be translated into benefits for human subjects in view of the different responses across species and even tissues (such as skeletal muscle specificity) to exercise? (4) Is there a cross talk between other proteogenic amino acids and leucine sensing and response in skeletal muscle, and what is the interaction mechanism? In future studies, the crucial upstream cascade of leucine sensing and response should be identified by means of bioinformatics or gene chip assay and should be further verified by using targeted gene knockout/overexpression animal models or blockers/agonists. Doing so will provide systematic theoretical and experimental clues for the application of related targets and mechanisms in the field of clinical and translational medicine.

## Author Contributions

YZ, JC, NZ, and ZX: substantial contributions to the conception or design of the work. YZ, HS, YY, XD, QW, and QS: drafting the work or revising it critically for important intellectual content. YZ, JC, NZ, and ZX: final approval of the version to be published. All authors agreed to be accountable for the content of the work.

## Conflict of Interest

The authors declare that the research was conducted in the absence of any commercial or financial relationships that could be construed as a potential conflict of interest.
